# Role of Latrophilin‐1 and Latrophilin‐2 as Downstream Effectors of Androgen Receptor Signaling in Urothelial Tumorigenesis

**DOI:** 10.1002/cnr2.70624

**Published:** 2026-07-15

**Authors:** Takuro Goto, Yuki Teramoto, Mohammad Amin Elahi Najafi, Masato Yasui, Gaku Yamamichi, Takuo Matsukawa, Hiroshi Miyamoto

**Affiliations:** ^1^ Department of Pathology & Laboratory Medicine University of Rochester Medical Center Rochester New York USA; ^2^ James P. Wilmot Cancer Institute University of Rochester Medical Center Rochester New York USA; ^3^ Department of Urology University of Rochester Medical Center Rochester New York USA; ^4^ Department of Pathology Johns Hopkins University School of Medicine Baltimore Maryland USA

**Keywords:** ADGRL1, ADGRL2, androgen receptor, LPHN1, LPHN2, urothelial cancer

## Abstract

**Background:**

Accumulating evidence indicates a critical role of androgen receptor (AR) in the pathogenesis of male‐dominant urothelial cancer. Meanwhile, the functions of latrophilins (LPHNs), a subfamily of G protein‐coupled receptors, remain largely uncharacterized in neoplastic conditions.

**Aims:**

To determine the functional role of LPHN1 and LPHN2 in association with AR signaling in urothelial tumorigenesis.

**Methods and Results:**

AR overexpression in immortalized human normal urothelial SVHUC cells or androgen treatment in SVHUC‐AR cells markedly increased the expression levels of LPHN1 and LPHN2. Chromatin immunoprecipitation assay demonstrated that AR could bind to the promoter regions of their encoded genes, *ADGRL1* and *ADGRL2*. In SVHUC‐AR cells exposed to a chemical carcinogen 3‐methylcholanthrene to induce neoplastic transformation, shRNA‐mediated knockdown of LPHN1 or LPHN2 significantly reduced their oncogenic activity, as measured by the ability of the resultant cells to form colonies and migrate. Immunohistochemistry in surgical specimens further revealed significantly higher levels of LPHN1 and LPHN2 expression in non‐muscle‐invasive bladder tumors than in adjacent non‐neoplastic urothelial tissues.

**Conclusions:**

The present findings suggest that LPHN1 and LPHN2 function as downstream mediators of AR signaling and promote urothelial tumorigenesis.

## 1 Introduction

Urinary bladder cancer, predominantly classified histologically as urothelial carcinoma, remains one of the most common malignancies, especially in male populations. Notably, the worldwide number of newly identified cases has substantially increased, from an estimated 429 800 in 2012 [1] to 613 791 reported in 2022 [2]. Bladder tumor is often detected as a nonfatal, non‐invasive disease, but affected patients face a substantial risk for postoperative recurrence and, in some cases, subsequent progression to invasive disease despite existing intravesical pharmacotherapy. Accordingly, identifying key molecular factors and signaling pathways involved in urothelial carcinogenesis is anticipated to facilitate the development of novel targeted strategies for the prevention of de novo or recurrent bladder tumors.

In addition to established exogenous risk factors, emerging evidence implicates the androgen receptor (AR), a member of the nuclear receptor superfamily, as an intrinsic contributor to the pathogenesis of urothelial cancer [3, 4, 5]. This involvement may particularly help explain the marked sex disparity observed in the incidence of bladder cancer. Sex‐related differences have additionally been identified in the dysregulation of other genes, including *KDM6A* [6] and members of the gonadotropin‐releasing hormone gene family such as *GNRH1* and *GNRHR* [7]. Nevertheless, the precise molecular mechanisms for how AR promotes urothelial tumorigenesis remain incompletely understood.

Latrophilins (LPHNs) comprise a subgroup of G protein‐coupled receptors initially isolated as binding partner(s) for latrotoxin (LTX), a neurotoxin component of the venom from black widow spider (e.g., *Latrodectus genus*) [8, 9, 10]. Among the three human isoforms, LPHN2 has been shown to be ubiquitously expressed, whereas LPHN1 and LPHN3 are abundant particularly in the brain [10, 11, 12]. Despite this knowledge, the precise biological functions of LPHNs and their encoded genes (i.e., *ADGRLs*) are largely undefined or untested, particularly in the context of neoplastic diseases.

Notably, *ADGRL3* was identified as one of the genes significantly down‐regulated in an AR‐knockdown bladder cancer subline, compared with control AR‐positive UMUC3 cells, in our prior DNA microarray analysis [13]. More recently, we provided preclinical evidence suggesting that androgen‐mediated AR signaling could up‐regulate the expression of *ADGRL3*/LPHN3 in urothelial cells and thereby induced the development of urothelial cancer [14]. We have additionally demonstrated that LPHN1 [15], LPHN2 [15], and LPHN3 [16] promote bladder cancer progression, a biological process considered distinct from cancer development (i.e., cancer initiation, malignant transformation, carcinogenesis). The present study was designed to determine whether LPHN1 and LPHN2 could contribute specifically to the malignant transformation of urothelial cells via serving as downstream mediators of AR signaling.

## 2 Methods

### 2.1 Cell Lines

The immortalized human normal urothelial cell line, SVHUC, was originally acquired from the American Type Culture Collection and then authenticated by the institutional core facility. Stable SVHUC sublines expressing either full‐length wild‐type human AR (i.e., SVHUC‐AR) or empty vector (i.e., SVHUC‐control), together with non‐silencing control short hairpin RNA (shRNA) (i.e., SVHUC‐control‐shRNA, SVHUC‐AR‐control‐shRNA), were established in our earlier investigations [17, 18]. In parallel, lentiviral particles carrying LPHN1‐shRNA (sc‐45408‐V, Santa Cruz Biotechnology) or LPHN2‐shRNA (sc‐60919‐V, Santa Cruz Biotechnology) were introduced into SVHUC (i.e., SVHUC‐LPHN1‐shRNA, SVHUC‐LPHN2‐shRNA) and SVHUC‐AR (i.e., SVHUC‐AR‐LPHN1‐shRNA, SVHUC‐AR‐LPHN2‐shRNA). All these SVHUC‐derived sublines were maintained in Ham's F‐12K (Thermo Fisher Scientific). For androgen stimulation experiments, cells were cultured in phenol red‐free medium supplemented with 5% charcoal‐stripped fetal bovine serum (FBS), whereas 5% FBS was used for all other experiments.

### 2.2 Chemicals and Antibodies

We obtained 3‐methylcholanthrene (MCA) from Sigma‐Aldrich, a synthetic androgen methyltrienolone (R1881) from PerkinElmer, α‐LTX from Alomone Labs, and recombinant human FLRT3 protein from R&D Systems. Primary antibodies, all purchased from Santa Cruz Biotechnology, included AR (clone 441), LPHN1 (clone A‐4), LPHN2 (clone E‐3), and GAPDH (clone 6C5).

### 2.3 Western Blot

Total cellular proteins were isolated using RIPA lysis buffer supplemented with a protease and phosphatase inhibitor cocktail (Thermo Fisher Scientific). Equal quantities of proteins (30 μg) were resolved by 10% sodium dodecyl sulfate‐polyacrylamide gel electrophoresis and subsequently transferred electrophoretically onto polyvinylidene difluoride membrane (Thermo Fisher Scientific). The membranes were blocked with 0.03%–0.3% blotting‐grade blocker (Bio‐Rad) and incubated with a specific primary antibody (i.e., AR [dilution 1:1000], LPHN1 [dilution 1:100], LPHN2 [dilution 1:100], GAPDH [dilution 1:5000]) overnight at 4°C and a secondary antibody (anti‐mouse or anti‐rabbit IgG HRP‐linked antibody; Cell Signaling Technology) for 1 h at room temperature. Chemiluminescent signals visualized by a Clarity Western ECL Substrate (Bio‐Rad) were detected with a ChemiDoc MP imaging system (Bio‐Rad). The densitometry values were measured using ImageJ software (National Institutes of Health).

### 2.4 Chromatin Immunoprecipitation (ChIP) Assay

Putative AR binding regions within the *ADGRL1* or *ADGRL2* promoter were first identified, using LASAGNA‐Search 2.0 (https://biogrid‐lasagna.engr.uconn.edu/lasagna_search/). A ChIP assay was subsequently conducted, using the Magna ChIP kit (Sigma‐Aldrich) following protocols we described previously [19, 20]. Briefly, soluble chromatin extracted from the cell lysates was immunoprecipitated with either an anti‐AR antibody or normal mouse IgG (sc‐2025, Santa Cruz Biochemistry), and the recovered DNA fragments were analyzed by PCR, using the following primer sets: *ADGRL1* forward, 5′‐TGCACAACCCTTCCAGATCT‐3′; *ADGRL1* reverse, 5′‐TTTCCTTCCTTTCGCCTCCT‐3′; *ADGRL2* forward, 5′‐TTGTGTACCTGGCCACTAATA‐3′; and *ADGRL2* reverse, 5′‐AATGAGGGACAGCGCAA‐3′.

### 2.5 In Vitro Transformation Assay

An established in vitro neoplastic/malignant transformation model employing SVHUC‐derived cells exposed to a chemical carcinogen MCA [21] was utilized with minor modifications (see Figure 2B). Cells (2 × 10^6^/10‐cm dish) treated with 5 μg/mL MCA for 48 h (serum‐free for the first 24 h, followed by 1% FBS) were cultured in medium containing 5% FBS until near confluence. Subcultured cells (1:3 split ratio) underwent two additional cycles of 48‐h MCA exposure. These MCA‐exposed cells were then continuously cultured for 6 weeks prior to use in further assays.

### 2.6 MTT Assay

The MCA‐exposed cells (3–5 × 10^3^/well) seeded in 96‐well tissue culture plates were cultured for 72–120 h and subsequently incubated with 0.5 mg/mL MTT solution (Sigma‐Aldrich) in 100 μL medium for 4 h. The resulting formazan crystals were dissolved in dimethyl sulfoxide, and absorbance values were measured at 570 nm with background correction at 630 nm.

### 2.7 Clonogenic Assay

The MCA‐exposed cells (2 × 10^2^/well) were seeded in 12‐well tissue culture plates and maintained until visible colonies developed in control wells. Colonies were stained with 0.1% crystal violet, photographed, and quantified based on colony area, using ImageJ software.

### 2.8 Wound‐Healing Assay

The MCA‐exposed cells grown to ≥ 90% confluence in 35‐mm culture dishes were scratched using a 200‐μL pipette tip. Following wounding, the cells were incubated in serum‐free medium for 24 h. Migration was assessed by measuring the normalized cell‐free area (24 h/0 h) from captured images, using ImageJ software.

### 2.9 Immunohistochemistry

Sets of bladder tissue microarray (TMA) containing transurethral resection specimens had been previously constructed following institutional ethical approval [22]. All these patients had non‐muscle‐invasive bladder tumor without evidence of metastatic disease at the time of surgery performed at either the University of Rochester Medical Center or The Johns Hopkins Hospital. Immunostaining was performed, using a primary antibody against LPHN1 or LPHN2 (both at 1:100 dilution), according to the protocols we described previously [14, 22]. Stained sections were evaluated by a board‐certified pathologist (H.M.) blinded to specimen identity. The immunoreactive scores were determined by multiplying the proportion of immunoreactive cells (0–4: 0% = 0; 1%–10% = 1; 11%–50% = 2; 51%–80% = 3; 81%–100% = 4) by staining intensity (0–3: negative = 0; weak = 1; moderate = 2; strong = 3). Final scores were categorized as negative (0; 0–1), weakly positive (1+; 2–4), moderately positive (2+; 6–8), or strongly positive (3+; 9–12).

### 2.10 Public Database Analysis

The R2 Genomics Analysis and Visualization Platform (https://hgserver1.amc.nl/cgi‐bin/r2/main.cgi) was used to assess the prognostic significance of *ADGRL1* and *ADGRL2* expression in patients with non‐muscle‐invasive bladder cancer [23]. Patients who had carcinoma in situ only (*n* = 3) or muscle‐invasive disease (*n* = 16), as well as those who underwent cystectomy (*n* = 26), were excluded from the analysis. *p‐*values derived from optimized cutoffs were not adjusted for multiple testing and are presented for exploratory purposes only.

### 2.11 Statistical Analysis

Student's *t*‐test and Fisher's exact test (two‐tailed) were used to evaluate numerical and categorized data, respectively. A *p‐*value below 0.05 was considered indicative of statistical significance.

## 3 Results

### 3.1 Impact of Androgen/AR on LPHN1 and LPHN2

**FIGURE 1:**
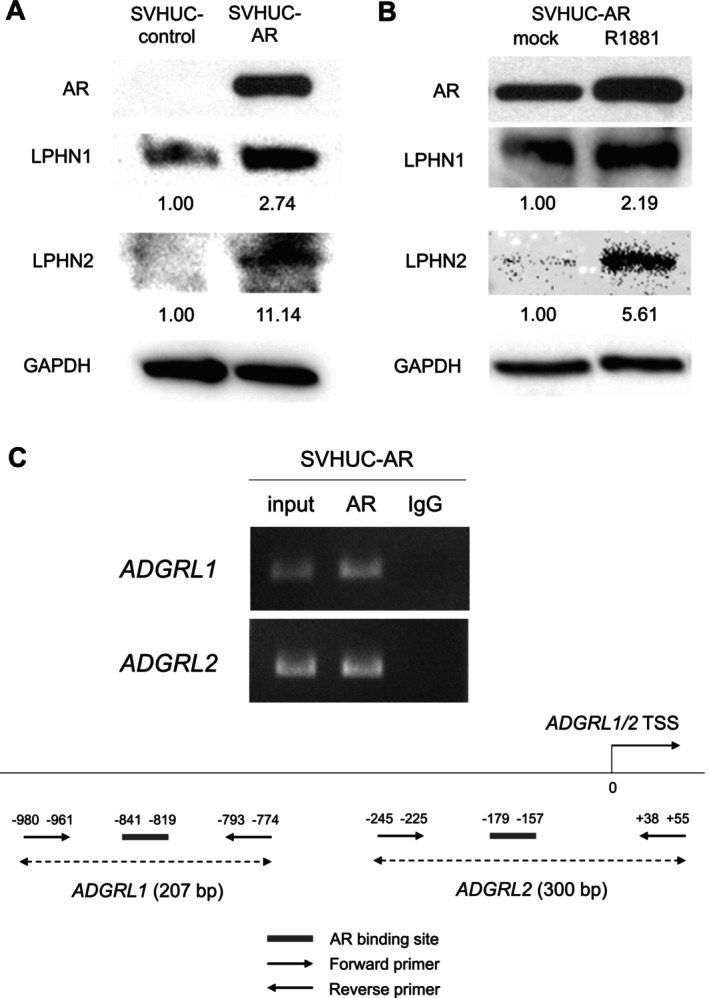
Associations between AR signaling and LPHN1/LPHN2 expression in urothelial cells. Western blotting of AR, LPHN1, and LPHN2 in SVHUC‐control vs. SVHUC‐AR (A) or SVHUC‐AR cultured for 48 h with ethanol (mock) or 10 nM R1881 (B). GAPDH served as a loading control. The densitometry values for LPHN1 and LPHN2, normalized by GAPDH and expressed relative to control cells (A) or mock treatment (B), are included below the lanes. (C) The ChIP assay, using SVHUC‐AR cell lysates immunoprecipitated with an anti‐AR (or IgG as a negative control). The DNA fragments were PCR amplified with sets of primers specific for the promoters of *ADGRL1* and *DGRL2*, and the PCR products were electrophoresed on 1% agarose gel. Fractions of the mixture of protein‐DNA complex (i.e., 1% of total cross‐linked, reserved chromatin prior to immunoprecipitation) were used as “input” DNAs.

We initially investigated the relationship between AR activity and LPHN expression in non‐neoplastic urothelial cells. Western blot demonstrated the substantially elevated levels of LPHN1 and LPHN2 in SVHUC‐AR relative to AR‐negative SVHUC‐control (Figure 1A). Additionally, in SVHUC‐AR, treatment with a synthetic androgen R1881 resulted in additional up‐regulation of LPHN1 and LPHN2 expression (Figure 1B).

A bioinformatics‐based search identified potential AR‐binding sites within the promoter regions of *ADGRL1* and *ADGRL2*, followed by validation using a ChIP assay (Figure 1C). DNA fragments immunoprecipitated from SVHUC‐AR cells with an anti‐AR antibody, but not with normal IgG, were successfully amplified by PCR with sets of primers targeting the predicted promoter regions of *ADGRL1* and *ADGRL2*.

### 3.2 Impact of LPHN1 and LPHN2 on the Neoplastic Transformation of Urothelial Cells

We next examined the effects of two known LPHN ligands, α‐LTX and FLRT3, on LPHN1/LPHN2 expression in urothelial cells. Western blot in SVHUC revealed that both ligands induced the expression of LPHN1 and LPHN2 (Figure S1).

**FIGURE 2:**
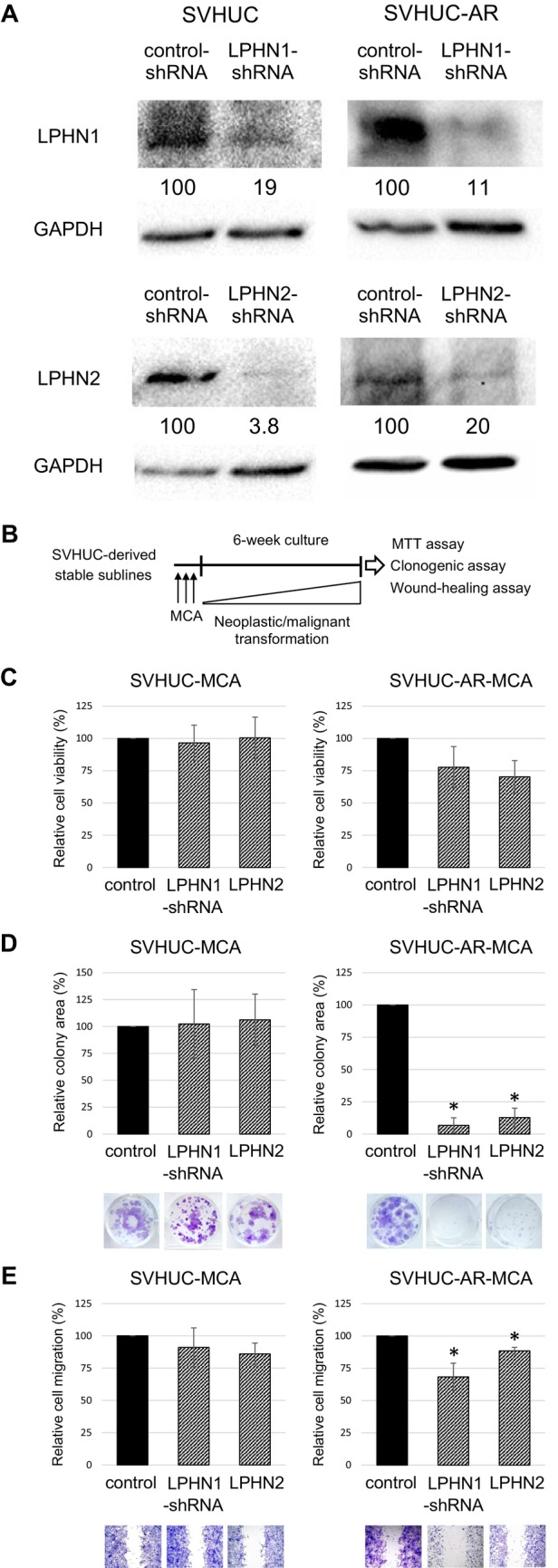
Effects of LPHN1/LPHN2 knockdown on the neoplastic transformation of urothelial cells. (A) Western blotting of LPHN1 and LPHN2 in SVHUC or AVHUC‐AR cells stably expressing control‐shRNA, LPHN1‐shRNA, or LPHN2‐shRNA. GAPDH served as a loading control. The densitometry values for LPHN1 and LPHN2, normalized by GAPDH and expressed relative to control‐shRNA cells, are included below the lanes. (B) A schema for in vitro transformation system. SVHUC‐control‐shRNA, SVHUC‐LPHN1‐shRNA, SVHUC‐LPHN2‐shRNA, SVHUC‐AR‐control‐shRNA, SVHUC‐AR‐LPHN1‐shRNA, and SVHUC‐AR‐LPHN2‐shRNA exposed to MCA and subsequently cultured for 6 weeks were seeded for MTT assay (C, additional 72–120‐h culture), clonogenic assay (D, additional 2‐week culture), or wound‐healing assay (E, additional 24‐h culture in serum‐free medium). Cell viability, colony area, or width of the wound area presented relative to that of control‐shRNA cells represents the mean (± SD) from three independent experiments. *p < 0.05 (vs. control‐shRNA subline via Student's *t*‐test).

We established stable knockdown sublines of SVHUC and SVHUC‐AR expressing LPHN1‐shRNA or LPHN2‐shRNA (Figure 2A). We then assessed the impact of LPHN1/LPHN2 expression on urothelial tumorigenesis, using an established MCA‐induced model in non‐neoplastic SVHUC‐derived cells known to gradually undergo neoplastic/malignant transformation during the course of subsequent 6‐week culture [18, 21]. Control (i.e., SVHUC‐control‐shRNA, SVHUC‐AR‐control‐shRNA) versus knockdown (i.e., SVHUC‐LPHN1‐shRNA, SVHUC‐LPHN2‐shRNA, SVHUC‐AR‐LPHN1‐shRNA, SVHUC‐AR‐LPHN2‐shRNA) sublines with three 48‐h MCA exposures were subcultured for 6 weeks (Figure 2B). The MCA‐mediated oncogenic activity in the transformed cells was finally measured by subsequent assays for cell viability (via MTT assay; Figure 2C), colony formation (via clonogenic assay; Figure 2D), and cell migration (via wound‐healing assay; Figure 2E). In AR‐negative SVHUC cells, knockdown of LPHN1 or LPHN2 did not significantly alter the degree of MCA‐induced transformation, as indicated by comparable growth behavior among resultant cells. In contrast, in MCA‐SVHUC‐AR cells characterized by higher basal levels of LPHN1/LPHN2 expression compared with SVHUC cells, LPHN1 or LPHN2 knockdown resulted in the significant inhibition of their neoplastic transformation, although their effects on the cell viability were modest.

### 3.3 Expression of LPHN1 and LPHN2 in Surgical Specimens

Immunohistochemical staining for LPHN1 and LPHN2 was performed in sets of bladder TMA comprising transurethral resection specimens from 75 patients with non‐muscle‐invasive bladder tumor. Positive immunoreactivity was observed in both the nucleus and cytoplasm of non‐neoplastic and neoplastic epithelial cells (Figure S2).

Semi‐quantitative analysis revealed that the expression levels of both LPHN1 and LPHN2 were significantly higher in bladder tumors than in paired non‐neoplastic normal‐appearing urothelial tissues (Table 1). Moreover, high‐grade (vs. low‐grade) tumors were significantly more often immunoreactive for LPHN1 (96.7% vs. 80%; *p* = 0.044) or LPHN2 (73.3% vs. 46.7%; *p* = 0.032). No significant differences in LPHN1 or LPHN2 expression were observed between bladder tumors from male versus female patients or between noninvasive pTa versus invasive pT1 diseases.

**TABLE 1:** Correlations of LPHN1 or LPHN2 expression in non‐muscle‐invasive bladder cancers with their clinicopathologic features.

	*N*	LPHN1 negative (0)	LPHN1 positive (1+)	LPHN1 positive (2+/3+)	*p* (0 vs. 1+/2+/3+)	*p* (0/1+ vs. 2+/3+)
Tissue					0.020	0.002
Normal urothelium	61	19 (31.1%)	29 (47.5%)	13 (21.3%)		
Urothelial tumor	75	10 (13.3%)	30 (40.0%)	35 (46.7%)		
Sex (normal)					1.000	1.000
Male	47	15 (31.9%)	22 (46.8%)	10 (21.3%)		
Female	14	4 (28.6%)	7 (50.0%)	3 (21.4%)		
Sex (tumor)					0.436	1.000
Male	56	9 (16.1%)	21 (37.5%)	26 (46.4%)		
Female	19	1 (5.3%)	9 (47.4%)	9 (47.4%)		
Tumor grade					0.044	0.167
Low^a^	45	9 (20.0%)	18 (40.0%)	18 (40.0%)		
High	30	1 (3.3%)	12 (40.0%)	17 (56.7%)		
pT stage					1.000	0.659
pTa^a^	70	10 (14.3%)	28 (40.0%)	32 (45.7%)		
pT1	5	0 (0%)	2 (40.0%)	3 (60.0%)		

	*N*	LPHN2 negative (0)	LPHN2 positive (1+)	LPHN2 positive (2+/3+)	*p* (0 vs. 1+/2+/3+)	*p* (0/1+ vs. 2+/3+)
Tissue					< 0.001	0.023
Normal urothelium	61	44 (72.1%)	16 (26.2%)	1 (1.6%)		
Urothelial tumor	75	32 (42.7%)	34 (45.3%)	9 (12.0%)		
Sex (normal)					0.738	1.000
Male	47	33 (70.2%)	13 (27.7%)	1 (2.1%)		
Female	14	11 (78.6%)	3 (21.4%)	0 (0%)		
Sex (tumor)					0.179	0.434
Male	56	21 (37.5%)	27 (48.2%)	8 (14.3%)		
Female	19	11 (57.9%)	7 (36.8%)	1 (5.3%)		
Tumor grade					0.032	0.470
Low^a^	45	24 (53.3%)	17 (37.8%)	4 (8.9%)		
High	30	8 (26.7%)	17 (56.7%)	5 (16.7%)		
pT stage					0.067	0.106
pTa^a^	70	32 (45.7%)	31 (44.3%)	7 (10.0%)		
pT1	5	0 (0%)	3 (60.0%)	2 (40.0%)		

^a^ Includes cases with papillary urothelial neoplasm of low malignant potential.

To further determine the prognostic relevance of *ADGRL1* and *ADGRL2* expression, we analyzed a publicly accessible dataset comprising a total of 431 patients with Ta or T1 bladder cancer. Data obtained from the E‐MTAB‐4321 cohort [23] revealed that high levels of *ADGRL1* (*p* = 0.028; Figure S3A) and *ADGRL2* (*p* = 0.043; Figure S3B) expression were associated with an increased risk of disease progression.

## 4 Discussion

G protein‐coupled receptors constitute a large and evolutionarily conserved protein superfamily that mediates a wide range of physiological and pathological processes [10, 24, 25]. However, evidence implicating LPHNs in malignant tumors remains relatively limited [10, 16, 26, 27, 28, 29]. Building on our recent demonstration indicating that LPHN3 promotes the development of urothelial cancer [14], the present study was designed to elucidate the functional role of LPHN1 and LPHN2 in the pathogenesis of urothelial cancer, with particular emphasis on their relationship to AR signaling, using an in vitro model for urothelial tumorigenesis.

Consistent with our previous findings for LPHN3, we observed that AR overexpression in non‐neoplastic urothelial SVHUC cells, as well as androgen treatment in SVHUC‐AR cells, considerably induced LPHN1 and LPHN2 expression. Furthermore, a ChIP assay in urothelial cells revealed binding of wild‐type AR to the promoter regions of *ADGRL1* and *ADGRL2*, supporting a direct regulatory interaction. Collectively, these results indicate that LPHN1 and LPHN2 represent direct downstream targets of AR signaling in urothelial cells. Nevertheless, additional studies, including reporter gene assays, will be required to definitely establish LPHN1 and LPHN2 as direct transcriptional targets of AR.

LTX, a neurotoxin present in the venom of widow spiders, has been shown to bind and activate all three members of the LPHN family [8, 9, 10]. FLRT3, a member of the fibronectin leucine rich transmembrane protein family, has additionally been identified as an endogenous ligand, at least for LPHN3 [10, 12, 30]. In urothelial SVHUC cells, we herein demonstrated that not only α‐LTX but also FLRT3 up‐regulated the expression of both LPHN1 and LPHN2.

It has been suggested that *ADGRL3*/LPHN3 contributes to the development of several types of malignancies, such as acute myeloid leukemia [27], breast cancer [28], and urothelial cancer [14], as a promoter. Altered exon inclusion in *ADGRL2* has also been documented in muscle‐invasive bladder cancers [31]. Using an in vitro system mediated by a chemical carcinogen MCA, we previously demonstrated that treatment with α‐LTX or FLRT3 strongly enhanced the neoplastic transformation of non‐neoplastic AR‐negative SVHUC cells and that LPHN3 knockdown resulted in its strong prevention in MCA‐SVHUC‐AR cells [14]. Moreover, in an in vivo model for bladder carcinogenesis, α‐LTX or FLRT3 treatment strongly accelerated the development of bladder cancers induced by a chemical carcinogen *N*‐butyl‐*N*‐(4‐hydroxybutyl)nitrosamine in female mice [14]. Correspondingly, the present study demonstrated that knockdown of LPHN1 or LPHN2 significantly inhibited the carcinogen‐induced neoplastic transformation of AR‐positive urothelial cells, as evidenced by decreased colony formation and migratory capacity, despite the modest inhibition of their viability. Interestingly, their knockdown exerted limited effects in AR‐negative cells possessing comparatively lower baseline LPHN1/LPHN2 expression (prior to knockdown). Together, these findings indicate that both LPHN1 and LPHN2 function as promoters of urothelial tumorigenesis. However, the downstream signaling pathways mediating LPHN‐driven oncogenic effects remain to be elucidated. It should also be acknowledged that the present study is limited by the lack of more contemporary preclinical models of urothelial carcinogenesis, including genetically engineered animals and patient‐derived organoids.

The expression profile of *ADGRL1*/LPHN1 and *ADGRL2*/LPHN2 in non‐neoplastic urothelial tissues versus urothelial tumors has not been well defined. Through immunohistochemical analysis in sets of TMA derived from transurethral resection specimens, we found that LPHN1/LPHN2 positivity (i.e., 0 vs. 1+/2+/3+) and LPHN1/LPHN2 overexpression (i.e., 0/1+ vs. 2+/3+) were significantly more often seen in non‐muscle‐invasive bladder tumors than in adjacent normal‐appearing urothelial tissues, supporting their oncogenic roles in urothelial cancer. Moreover, the positive rates of LPHN1 or LPHN2 expression were significantly higher in high‐grade tumors than in low‐grade tumors. In addition, analysis of a publicly available transcriptomic dataset [23] demonstrated an association between elevated *ADGRL1*/*ADGRL2* expression in non‐muscle‐invasive bladder cancers and less favorable oncologic outcomes. These immunohistochemical and RNA sequencing data obtained from surgical specimens strongly support our in vitro findings presented here and may further substantiate the oncogenic roles of LPHN1 and LPHN2 in urothelial tumorigenesis.

In conclusion, the present study identifies LPHN1 and LPHN2 as downstream effectors of AR signaling in urothelial cells that promote their tumorigenesis, whereas LPHNs have previously been implicated in bladder tumor progression [15, 16], a biologically distinct process. Together with our previous data on LPHN3 [14], overexpression and/or activation of the LPHN family might represent a key mechanistic link between AR signaling and bladder cancer development. Accordingly, pharmacologic inhibition of LPHN(s), either alone or in combination with anti‐AR therapy, may provide a promising chemopreventive approach for de novo urothelial cancer or postoperative recurrent superficial bladder tumor. Further investigation is warranted to not only validate our present results but also define the molecular pathways through which LPHNs drive urothelial tumorigenesis.

## Author Contributions

**Takuro Goto:** data curation, formal analysis, writing – original draft. **Yuki Teramoto:** data curation, writing – review and editing. **Mohammad Amin Elahi Najafi:** data curation, writing – review and editing. **Masato Yasui:** data curation, writing – review and editing. **Gaku Yamamichi:** writing – review and editing, data curation. **Takuo Matsukawa:** data curation, writing – review and editing. **Hiroshi Miyamoto:** conceptualization, supervision, funding acquisition, writing – review and editing, data curation.

## Funding

This work has been supported by Ferring Innovation Grants.

## Ethics Statement

Appropriate approval from the Institutional Review Boards at the University of Rochester Medical Center (RSRB00032588) and The Johns Hopkins Hospital (N0:03‐03‐07‐02d) before construction and use of the sets of bladder tissue microarray. All the procedures in this study were conducted in accordance with the guidelines of the Declaration of Helsinki.

## Consent

Informed consent was waived due to the retrospective nature of the study and the analysis of de‐identified data.

## Conflicts of Interest

The authors declare no conflicts of interest.

## Supporting information


**Figure S1:** Effects of ligand treatment on the expression of LPHNs.

**Figure S2:** Immunohistochemistry of LPHN1 and LPHN2 in bladder tissue microarray.

**Figure S3:** Kaplan–Meier curves for progression‐free survival, according to the levels of *ADGRL1* (A) and *ADGRL2* (B) expression in patients with Ta or T1 bladder cancer who did not undergo cystectomy.

## Data Availability

The data that support the findings of this study are available on request from the corresponding author. The data are not publicly available due to privacy or ethical restrictions.
