# Supplementary material for: Role of Latrophilin‐1 and Latrophilin‐2 as Downstream Effectors of Androgen Receptor Signaling in Urothelial Tumorigenesis
Source: Cancer Rep (Hoboken). 2026 Jul 15;9(7):e70624. doi: 10.1002/cnr2.70624 (PMC13370665; doi:10.1002/cnr2.70624)
Supplement: Supplementary file 1 — Figure S1: Effects of ligand treatment on the expression of LPHNs. [file CNR2-9-e70624-s003.pptx]

## Slide 1
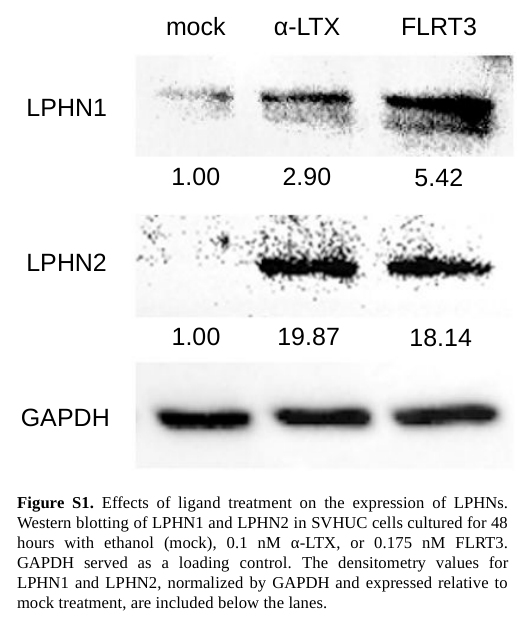

FLRT3
mock
α-LTX
LPHN1
1.00
2.90
5.42
LPHN2
1.00
19.87
18.14
GAPDH
Figure S1. Effects of ligand treatment on the expression of LPHNs. Western blotting of LPHN1 and LPHN2 in SVHUC cells cultured for 48 hours with ethanol (mock), 0.1 nM α-LTX, or 0.175 nM FLRT3. GAPDH served as a loading control. The densitometry values for LPHN1 and LPHN2, normalized by GAPDH and expressed relative to mock treatment, are included below the lanes.
