# Supplementary material for: Role of Latrophilin‐1 and Latrophilin‐2 as Downstream Effectors of Androgen Receptor Signaling in Urothelial Tumorigenesis
Source: Cancer Rep (Hoboken). 2026 Jul 15;9(7):e70624. doi: 10.1002/cnr2.70624 (PMC13370665; doi:10.1002/cnr2.70624)
Supplement: Supplementary file 2 — Figure S2: Immunohistochemistry of LPHN1 and LPHN2 in bladder tissue microarray. [file CNR2-9-e70624-s002.pptx]

## Slide 1
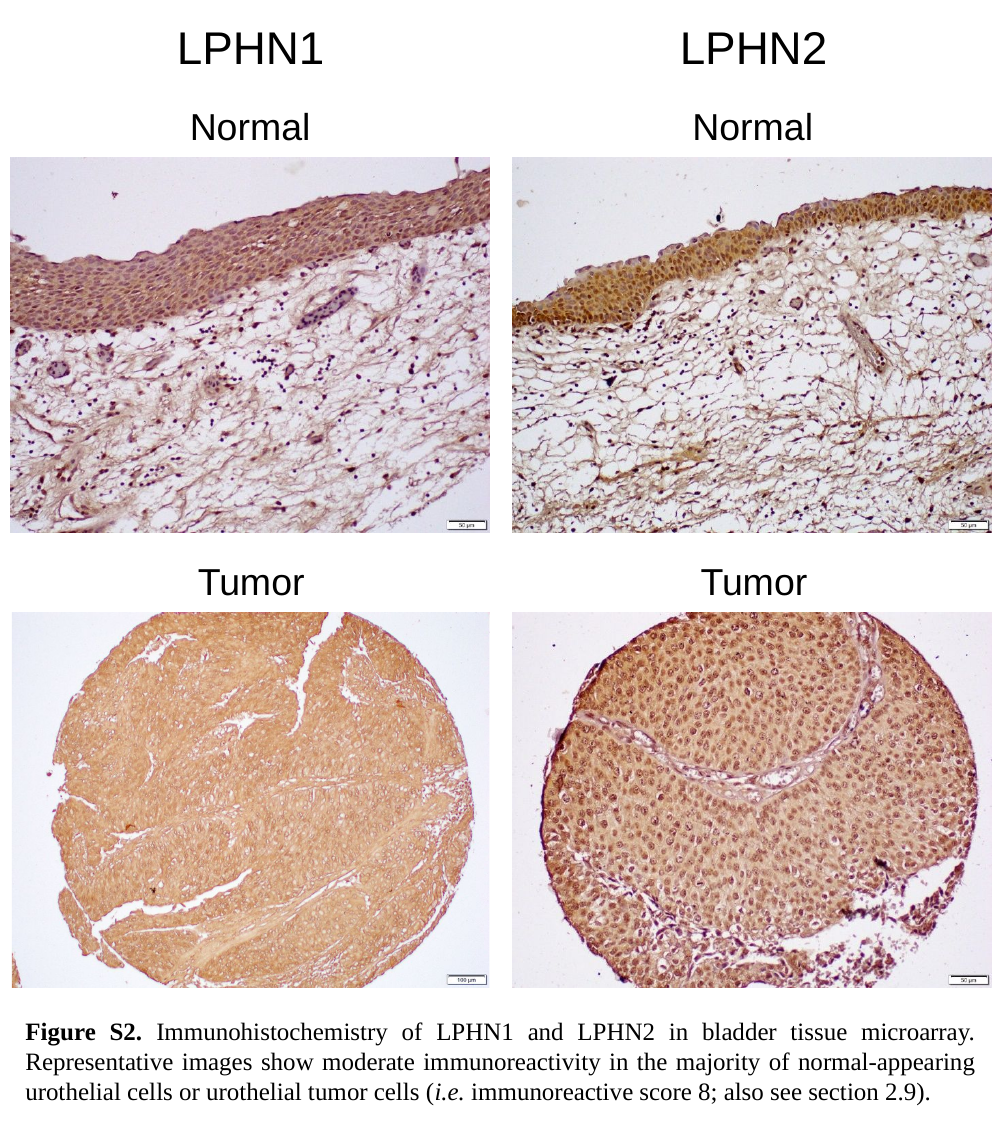

LPHN1
LPHN2
Normal
Normal
Tumor
Tumor
Figure S2. Immunohistochemistry of LPHN1 and LPHN2 in bladder tissue microarray. Representative images show moderate immunoreactivity in the majority of normal-appearing urothelial cells or urothelial tumor cells (i.e. immunoreactive score 8; also see section 2.9).
