# Supplementary material for: Role of Latrophilin‐1 and Latrophilin‐2 as Downstream Effectors of Androgen Receptor Signaling in Urothelial Tumorigenesis
Source: Cancer Rep (Hoboken). 2026 Jul 15;9(7):e70624. doi: 10.1002/cnr2.70624 (PMC13370665; doi:10.1002/cnr2.70624)
Supplement: Supplementary file 3 — Figure S3: Kaplan–Meier curves for progression‐free survival, according to the levels of ADGRL1 (A) and ADGRL2 (B) expression in patients with Ta or T1 bladder cancer who did not undergo cystectomy. [file CNR2-9-e70624-s001.pptx]

## Slide 1
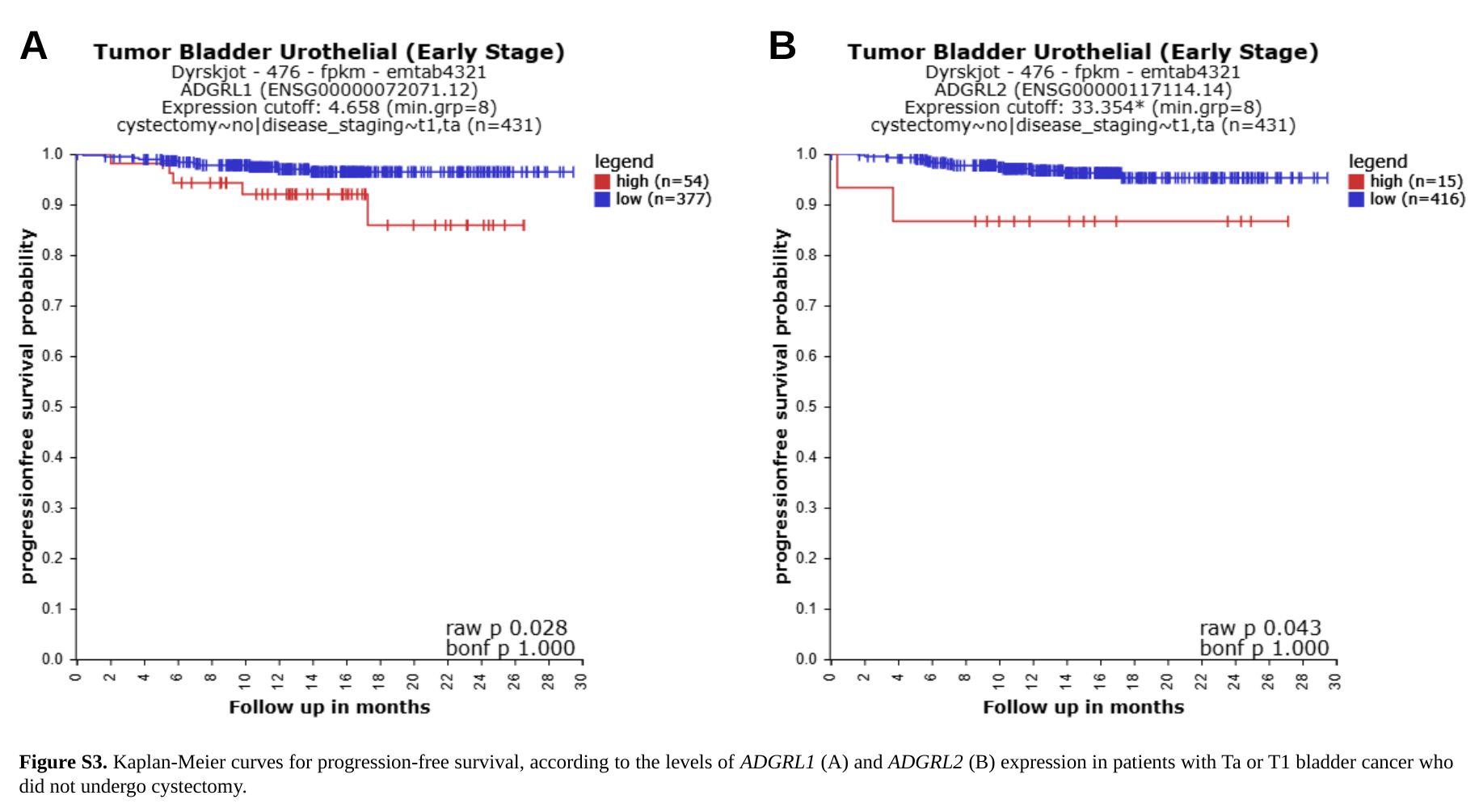

A
B
Figure S3. Kaplan-Meier curves for progression-free survival, according to the levels of ADGRL1 (A) and ADGRL2 (B) expression in patients with Ta or T1 bladder cancer who did not undergo cystectomy.
